# Supplementary material for: Effects of SPI1-mediated transcriptome remodeling on Alzheimer’s disease-related phenotypes in mouse models of Aβ amyloidosis
Source: Nat Commun. 2024 May 11;15:3996. doi: 10.1038/s41467-024-48484-x (PMC11088624; doi:10.1038/s41467-024-48484-x)
Supplement: Supplementary file 1 — Supplementary Information [file 41467_2024_48484_MOESM1_ESM.pdf]

# Supplementary Figure 1

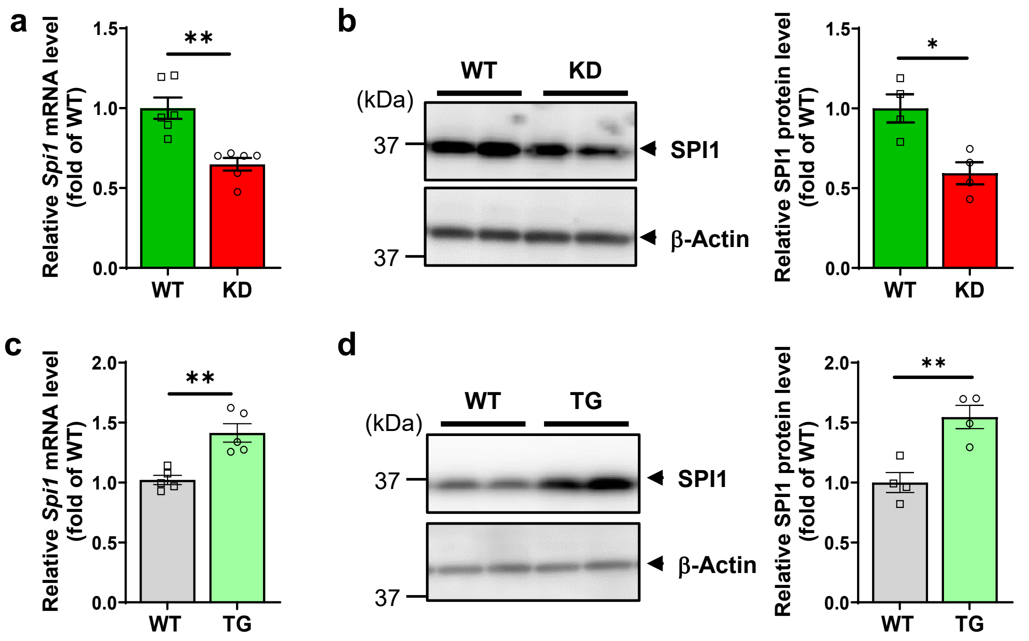


**Supplementary Fig. 1 | *Spi1* expression levels in *Spi1* mutant mice compared to each littermate-control. a,** The qPCR analysis of *Spi1* mRNA level in the cortex of two-month-old male *Spi1*-wildtype (WT) and *Spi1*-knockdown (KD) mice. All values are mean ± SEM. $^{**}p<0.01$(unpaired, two-tailed *t*-test; $n$ = 6 per group). **b,** The WB analysis of SPI1 protein levels in cortex of two-month-old male WT and KD mice. β-actin was used as the loading control. All values are mean ± SEM. $^{*}p<0.05$ (unpaired, two-tailed *t*-test; $n$ = 4 per group).

**c,** The qPCR analysis of *Spi1* mRNA level in the cortex of two-month-old male *Spi1* WT and *Spi1*-transgenic (TG) mice. All values are mean ± SEM. $^{**}p<0.01$(unpaired, two-tailed *t*-test; $n$ = 5 per group). **d,** The WB analysis of SPI1 protein levels in cortex of two-month-old male WT and TG mice. β-actin was used as the loading control. All values are mean ± SEM. $^{**}p<0.01$ (unpaired, two-tailed *t*-test; $n$ = 4 per group). Source data are provided as a Source data file.

# Supplementary Figure 2

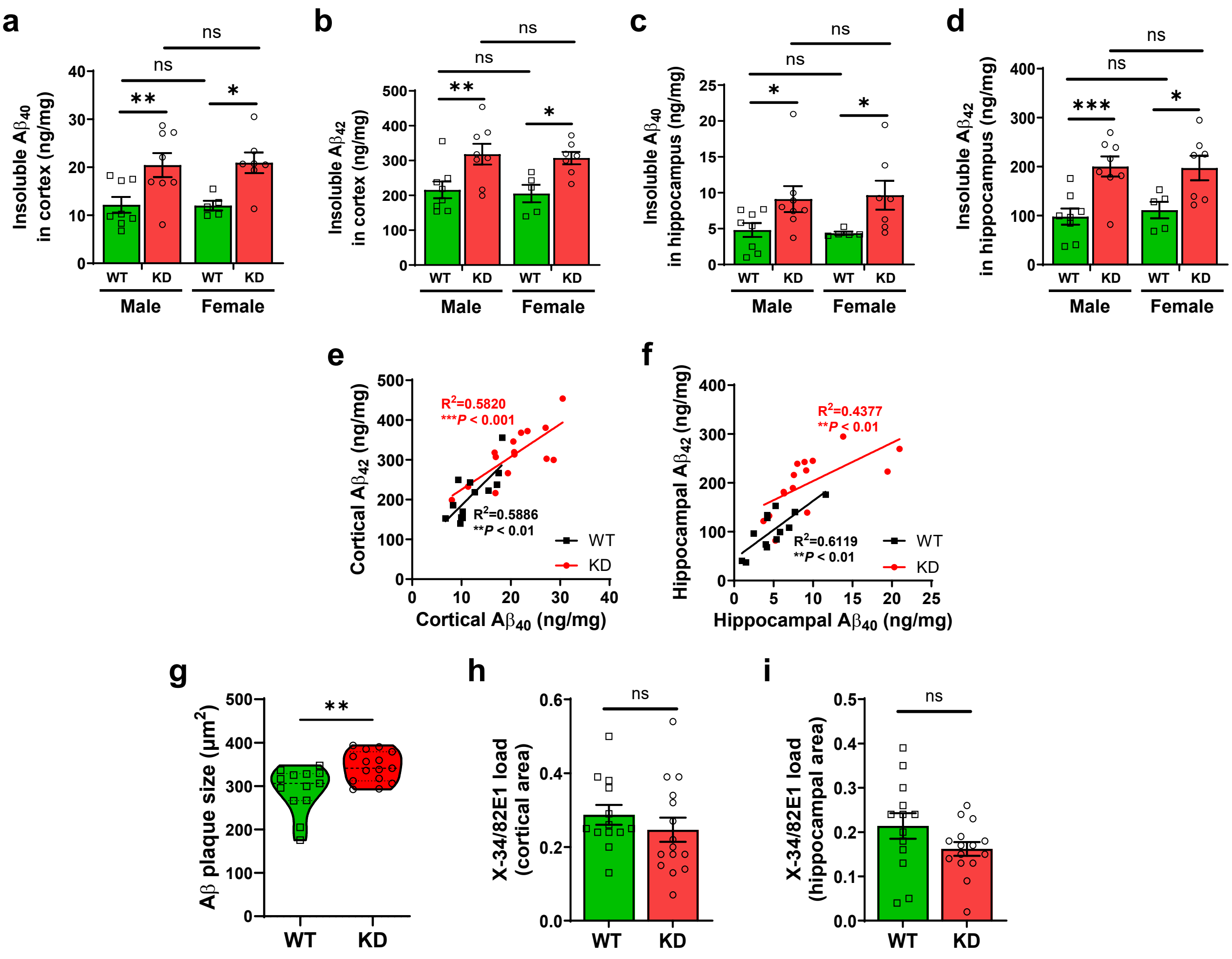


**Supplementary Fig. 2 | Reducing *Spi1* expression has no differential sex effect on $A\beta_{40}$ and $A\beta_{42}$ levels in the cortex and hippocampus of APP/PS1 mice. a-d,** The MSD Aβ ELISA analysis of $A\beta_{40}$ (**a,c**) and $A\beta_{42}$ (**b,d**) levels from the guanidine fraction of the cortex (**a-b**) and hippocampus (**c-d**) of *Spi1*$^{+/+}$;APP/PS1 mice and *Spi1*$^{+/-}$;APP/PS1 mice. All values are mean ± SEM. *$p$<0.05, **$p$<0.01, and ***$p$<0.001 (two-way ANOVA with *Ficher's* LSD multiple comparisons test; WT, $n$ = 8 for male, $n$ = 5 for female; KD, $n$ = 8 for male, $n$ = 7 for female; *ns*, not significant). **e-f,** Correlation between the insoluble $A\beta_{40}$ and $A\beta_{42}$ levels in the cortex (**e**) and hippocampus (**f**) of *Spi1*$^{+/+}$;APP/PS1 mice and *Spi1*$^{+/-}$;APP/PS1 mice. **$p$<0.01 and ***$p$<0.001 (*Pearson's* correlation coefficients analysis; WT, $n$ = 13 for *Spi1*$^{+/+}$;APP/PS1; KD, $n$ = 15 for *Spi1*$^{+/-}$;APP/PS1). **g,** Quantitation of the size of Aβ plaques in the cortex of *Spi1*$^{+/+}$;APP/PS1 mice and *Spi1*$^{+/-}$;APP/PS1 mice. **$p$<0.01 (unpaired, two-tailed *t*-test; WT, $n$ = 13 for *Spi1*$^{+/+}$;APP/PS1; KD, $n$ = 15 for *Spi1*$^{+/-}$;APP/PS1). **h,i,** The ratio of fibrillar plaque to total plaque load in the cortex (**h**) and hippocampus (**i**) of *Spi1*$^{+/+}$;APP/PS1 mice and *Spi1*$^{+/-}$;APP/PS1 mice. All values are mean ± SEM. (unpaired, two-tailed *t*-test; WT, $n$ = 13 for *Spi1*$^{+/+}$;APP/PS1; KD, $n$ = 15 for *Spi1*$^{+/-}$;APP/PS1; *ns*, not significant). Source data are provided as a Source data file.

# Supplementary Figure 3

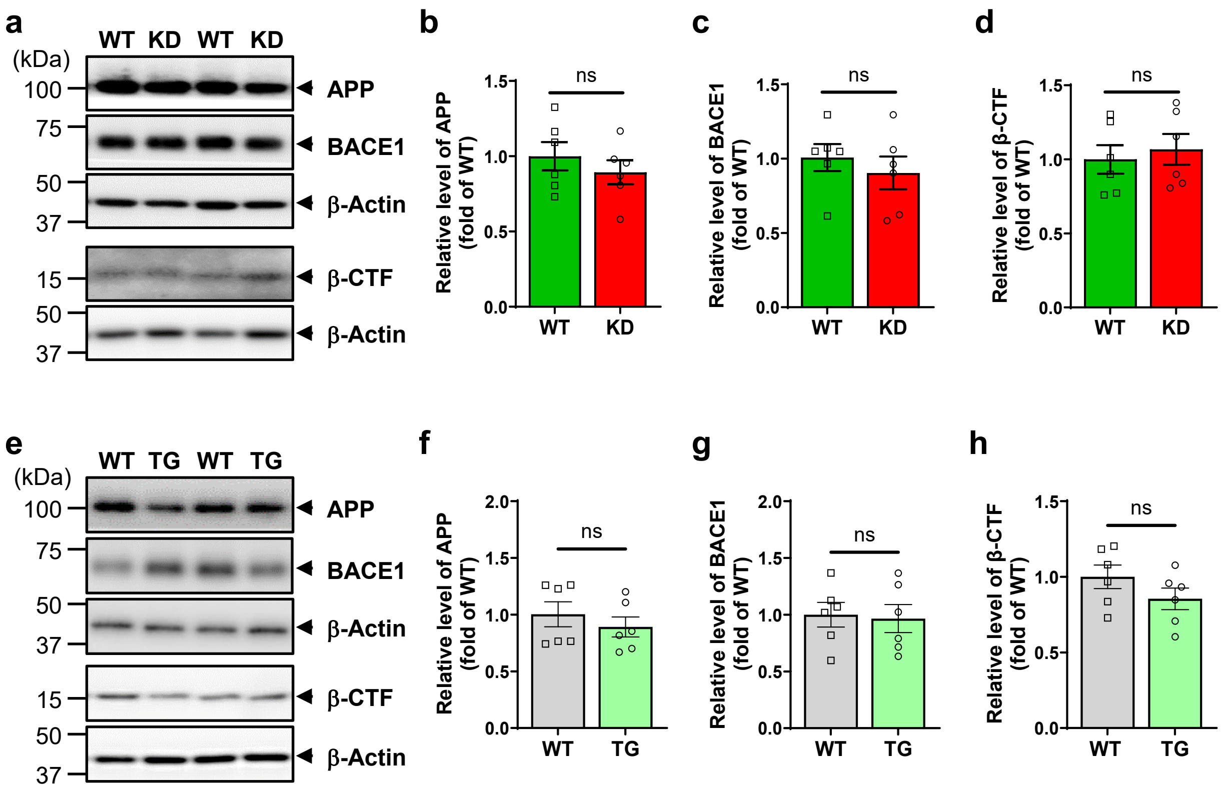


**Supplementary Fig. 3 | Knockdown of *Spi1* does not affect full-length APP and BACE1 protein levels. a-d,** Western blot analyses of APP, BACE1, and β-CTF in the cortex of *Spi1*$^{+/+}$;APP/PS1 (WT) mice and *Spi1*$^{+/-}$;APP/PS1(KD) mice. **a,** Representative images of western blots. Quantification of the relative protein levels of APP (**b**), BACE1 (**c**), and β-CTF (**d**) immunoblots. Data were normalized by β-actin levels and expressed as fold-change relative to WT. All values are mean ± SEM. (unpaired, two-tailed *t*-test; *n* = 6 for each group; *ns*, not significant).
**Overexpression of *Spi1* does not affect full-length APP and BACE1 protein levels. e-h,** Western blot analyses of APP, BACE1, and β-CTF in the cortex of *Spi1*$^{+/+}$;5XFAD (WT) and *Spi1*$^{Tg/0}$;5XFAD (TG) mice. **e,** Representative images of western blots. Quantification of the relative protein levels of APP (**f**), BACE1 (**g**), and β-CTF (**h**) immunoblots. Data were normalized by β-Actin levels and expressed as fold-change relative to WT. All values are mean ± SEM. (unpaired, two-tailed *t*-test; *n* = 6 for each group; *ns*, not significant). Source data are provided as a Source data file.

# Supplementary Figure 4

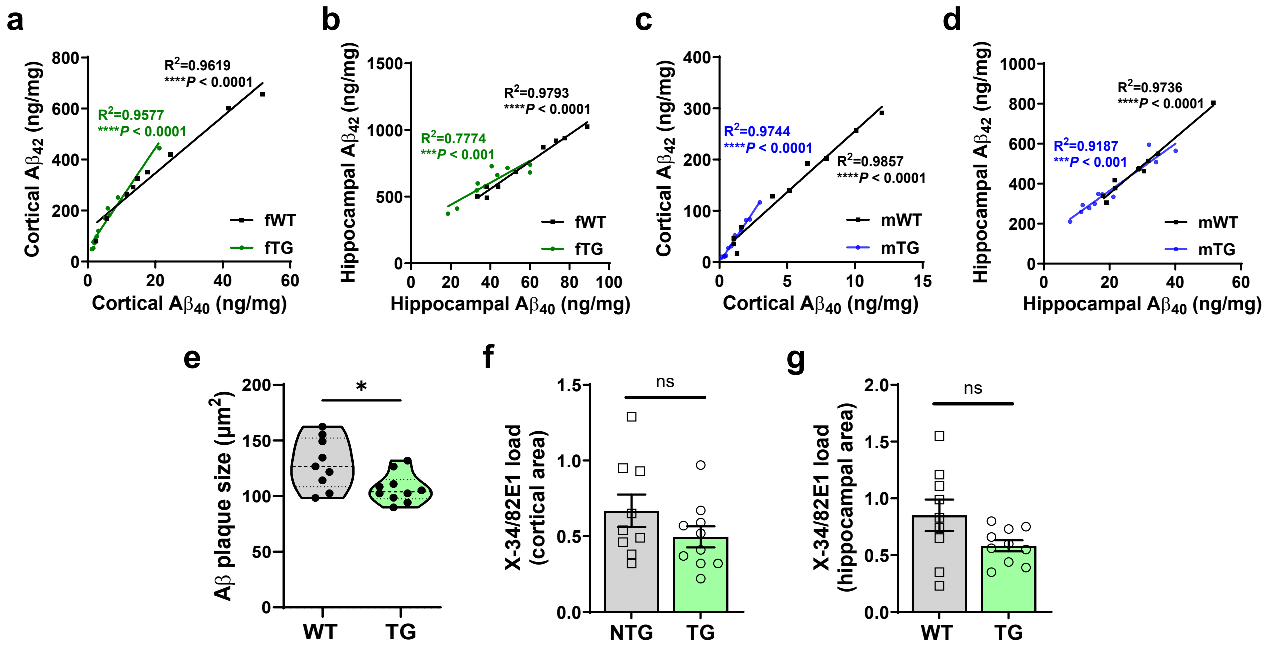


**Supplementary Fig. 4 | Insoluble $A\beta_{40}$ and $A\beta_{42}$ levels have a strong correlation in the cortex and hippocampus of 5XFAD mice. a-b,** Correlation of the insoluble $A\beta_{40}$ and $A\beta_{42}$ levels in the cortex (**a**) and hippocampus (**b**) of female *Spi1*$^{+/+}$;5XFAD (fWT) and *Spi1*$^{Tg/0}$;5XFAD (fTG) mice. ***$p$<0.001 and ****$p$<0.0001 (*Pearson's* correlation coefficients analysis; fWT, $n$ = 9 for *Spi1*$^{+/+}$;5XFAD; fTG, $n$ = 10 for *Spi1*$^{Tg/0}$;5XFAD). **c-d,** Correlation of the insoluble $A\beta_{40}$ and $A\beta_{42}$ levels in the cortex (**c**) and hippocampus (**d**) of male *Spi1*$^{+/+}$;5XFAD (mWT) and *Spi1*$^{Tg/0}$;5XFAD (mTG) mice. ***$p$<0.001 and ****$p$<0.0001 (*Pearson's* correlation coefficients analysis; mWT, $n$ = 10 for *Spi1*$^{+/+}$;5XFAD; mTG, $n$ = 11 for *Spi1*$^{Tg/0}$;5XFAD). **e,** Quantitation of the size of Aβ plaques in the cortex of *Spi1*$^{+/+}$;5XFAD and *Spi1*$^{Tg/0}$;5XFAD mice. *$p$<0.05 (unpaired, two-tailed $t$-test; WT, $n$ = 9 for *Spi1*$^{+/+}$;5XFAD; TG, $n$ = 10 for *Spi1*$^{Tg/0}$;5XFAD). **f,g,** The ratio of fibrillar plaque to total plaque load in the cortex (**f**) and hippocampus (**g**) of *Spi1*$^{+/+}$;5XFAD mice and *Spi1*$^{Tg/0}$;5XFAD mice. All values are mean ± SEM. (unpaired, two-tailed $t$-test; WT, $n$ = 9 for *Spi1*$^{+/+}$;5XFAD; TG, $n$ = 10 for *Spi1*$^{Tg/0}$;5XFAD; *ns*, not significant). Source data are provided as a Source data file.

# Supplementary Figure 5

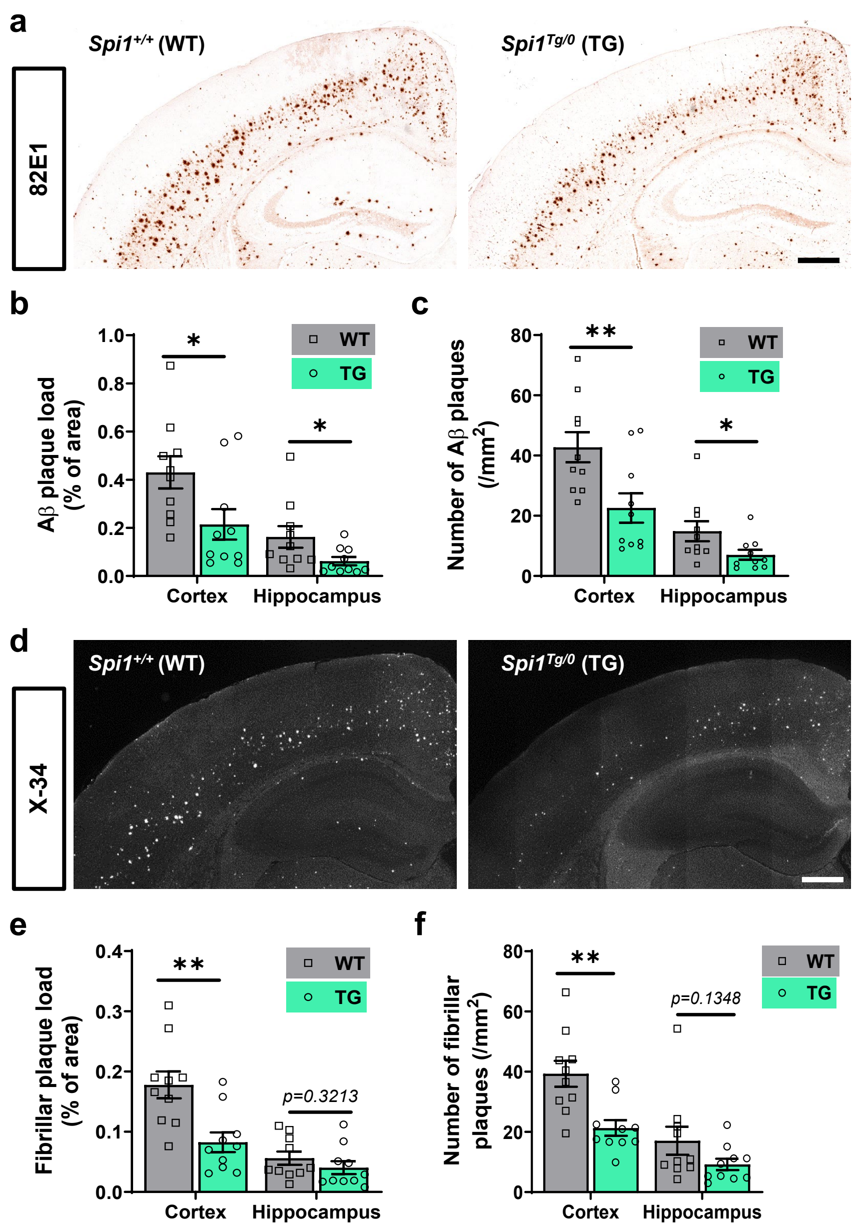


**Supplementary Fig. 5 | *Spi1*-overexpression decreases 82E1-positive Aβ plaques and X-34-positive fibrillar plaques in the brains of male 5XFAD mice. a,** Representative images of 82E1-positive plaques in brain slices from male *Spi1*$^{+/+}$;5XFAD and *Spi1*$^{Tg/0}$;5XFAD mice. *Scale bars*, 500 μm. **b-c,** Quantification of the Aβ plaque deposition (**b**) and the total number of Aβ plaques (**c**) in the cortical and hippocampal areas. All values are mean ± SEM. *$p<0.05$ and **$p<0.01$ (unpaired, two-tailed *t*-test; WT, $n$ = 10 for *Spi1*$^{+/+}$;5XFAD; TG, $n$ = 10 for *Spi1*$^{Tg/0}$;5XFAD). **d,** Representative images of X-34-positive fibrillar plaque in brain slices from male *Spi1*$^{+/+}$;5XFAD and *Spi1*$^{Tg/0}$;5XFAD mice. *Scale bars*, 500 μm. **e-f,** Quantification of the fibrillar plaque deposition (**e**), and the total number of fibrillar plaques (**f**) in the cortical and hippocampal areas. All values are mean ± SEM. **$p<0.01$ (unpaired, two-tailed *t*-test; WT, $n$ = 10 for *Spi1*$^{+/+}$;5XFAD; TG, $n$ = 10 for *Spi1*$^{Tg/0}$;5XFAD). Source data are provided as a Source data file.

# Supplementary Figure 6

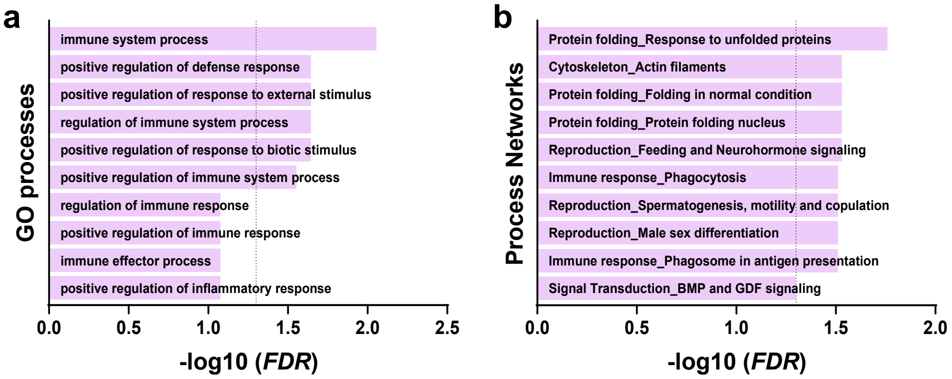


**Supplementary Fig. 6 | Integrative analysis of DEG in *Spi1*-knockdown and -overexpression mouse models. a-b,** GO process enrichment analysis (**a**) and Process Networks enrichment analysis (**b**) with the functional annotation of DEGs from knockdown and overexpression data were performed using the MetaCore software. *P*-values of **a**,**b** were calculated using Metacore algorithms with threshold of significant enrichment as adjusted $p<0.05$ (shown as a vertical dash line for **a,b**). Source data are provided as Supplementary information.

# Supplementary Figure 7

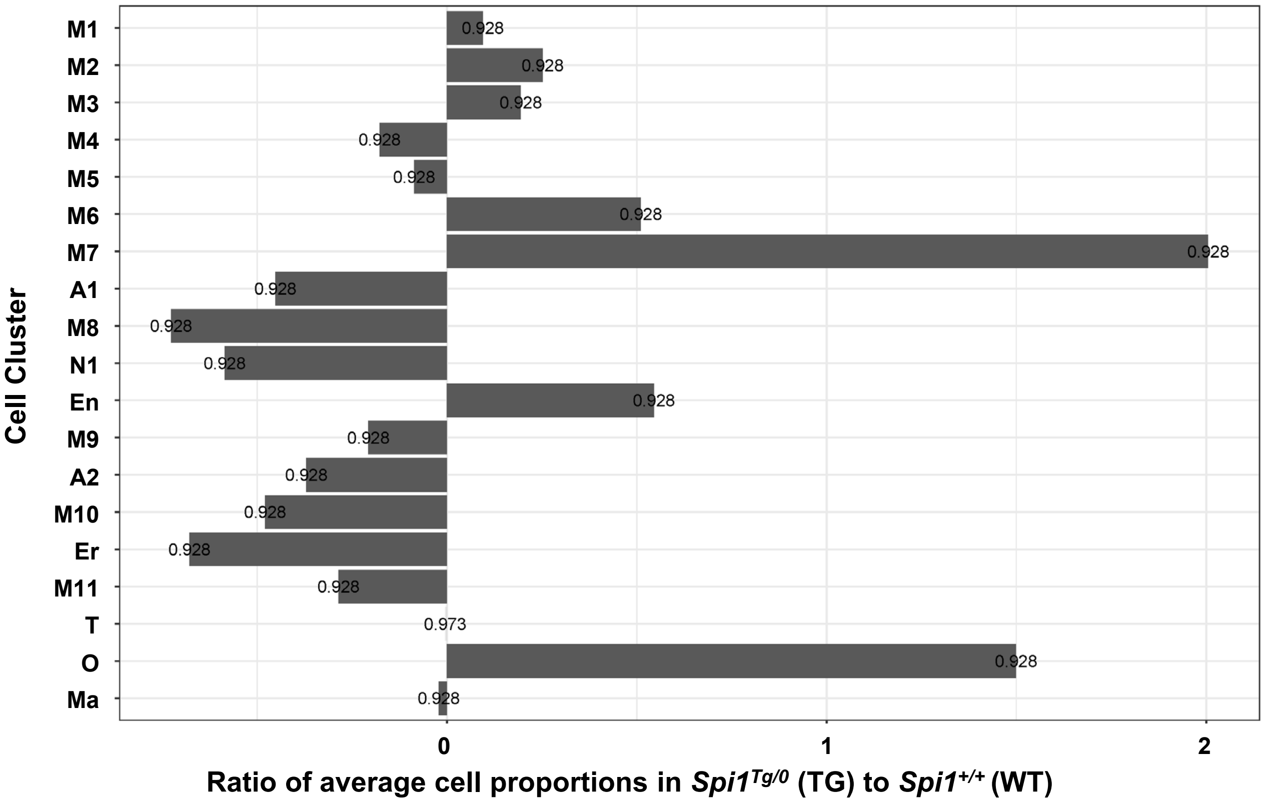


**Supplementary Fig. 7 | Ratio of average cell proportions per cluster per genotype.** Shown are bar plots per each cell cluster (M1-11=microglia; A1-2=astrocytes; En=endothelial cells; Er=erythrocytes; Ma=macrophages; N1=neurons; O=oligodendrocytes; and T=T-cells) representing the ratio of average cell proportions between the genotypes (Cells in *Spi1*$^{Tg/0}$;5XFAD mice/Cells in *Spi1*$^{+/+}$;5XFAD mice). *Benjamini-Hochberg*-adjusted *p*-values are shown at the end of each bar. Source data are provided as a Source data file.

## Supplementary Figure 8

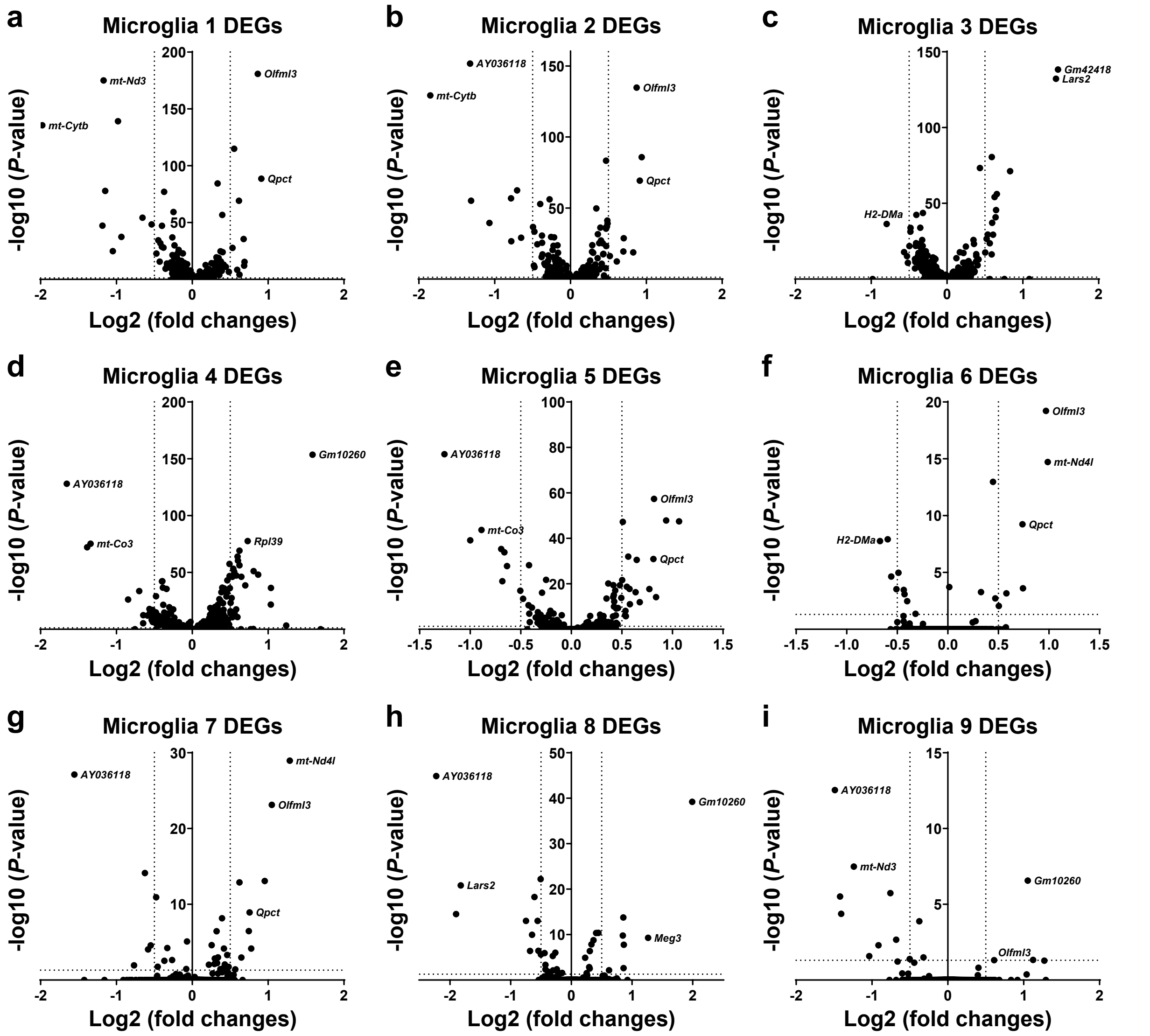


**Supplementary Fig. 8 | scRNA-seq identified DEGs in microglia clusters in *Spi1*$^{+/+}$;5XFAD and *Spi1*$^{Tg/0}$;5XFAD mice. a-i,** Volcano plot showing DEGs in microglia clusters 1-9. The dashed lines on the volcano plots represent significance as the *Benjamini-Hochberg*-adjusted -log10 *p*-value, where adjusted *p*<0.05 (y-axis) and a log2 fold change of 0.585 (x-axis). Source data are provided as Supplementary information.

# Supplementary Figure 9

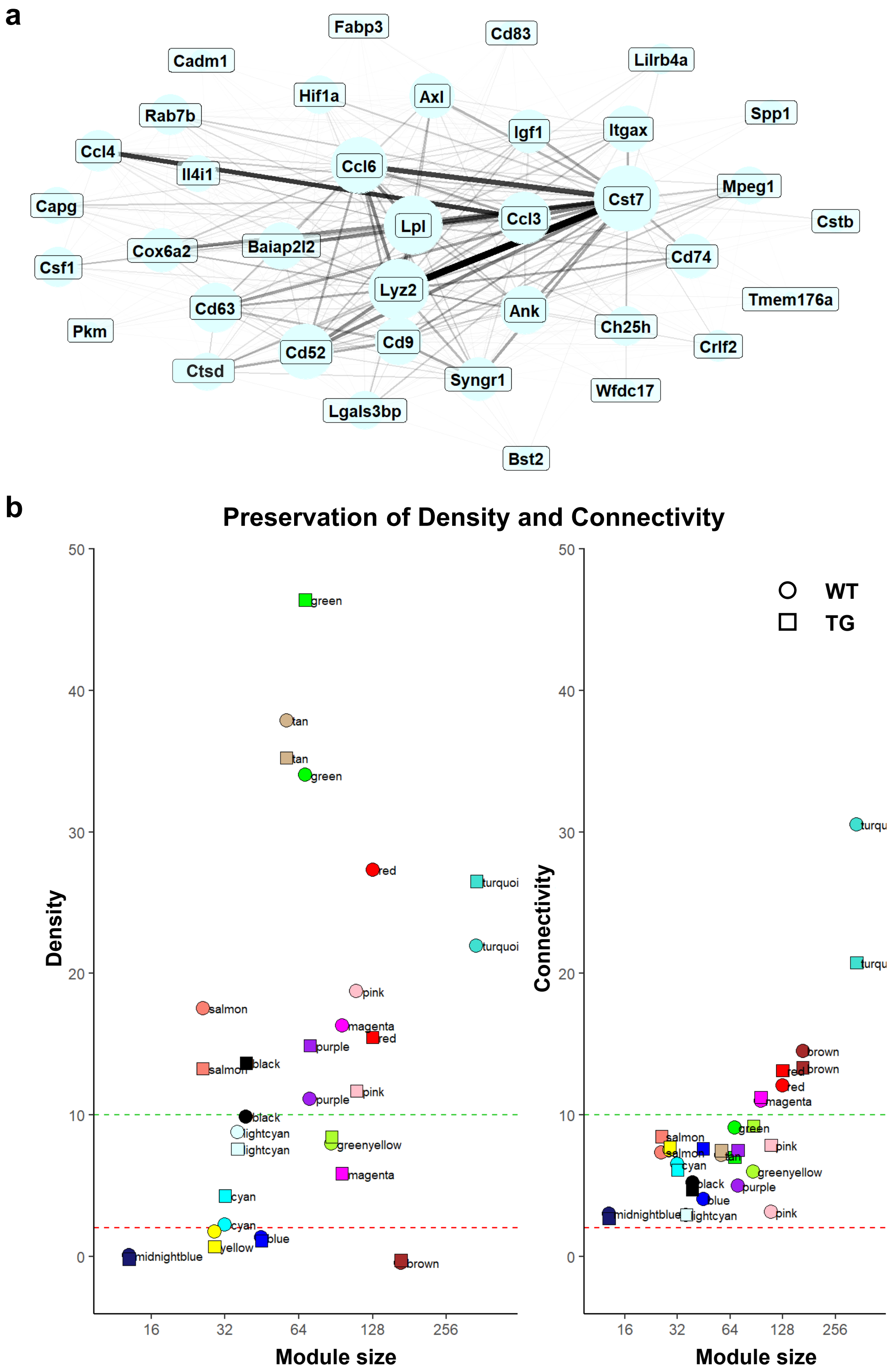


**Supplementary Fig. 9 | scWGCNA identified a DAM-like gene network in microglial cluster 4. a,** The network genes and features of the light cyan module, which were enriched across microglial cluster 4. Connection opacity denotes interaction weight, and node size denotes centrality within the module measured by Maximal Clique Centrality. **b,** Comparison of module preservation did not reveal a significant difference in density (how intact the network is) or connectivity (how similar the network connectivity is) for any module between *Spi1*$^{+/+}$;5XFAD (WT) and *Spi1*$^{Tg/0}$;5XFAD (TG) mice. For both plots, density and connectivity summary statistic thresholds of Z=2 and Z=10 are shown as red and green dashed lines, respectively. Source data are provided as Supplementary information.

# Supplementary Figure 10

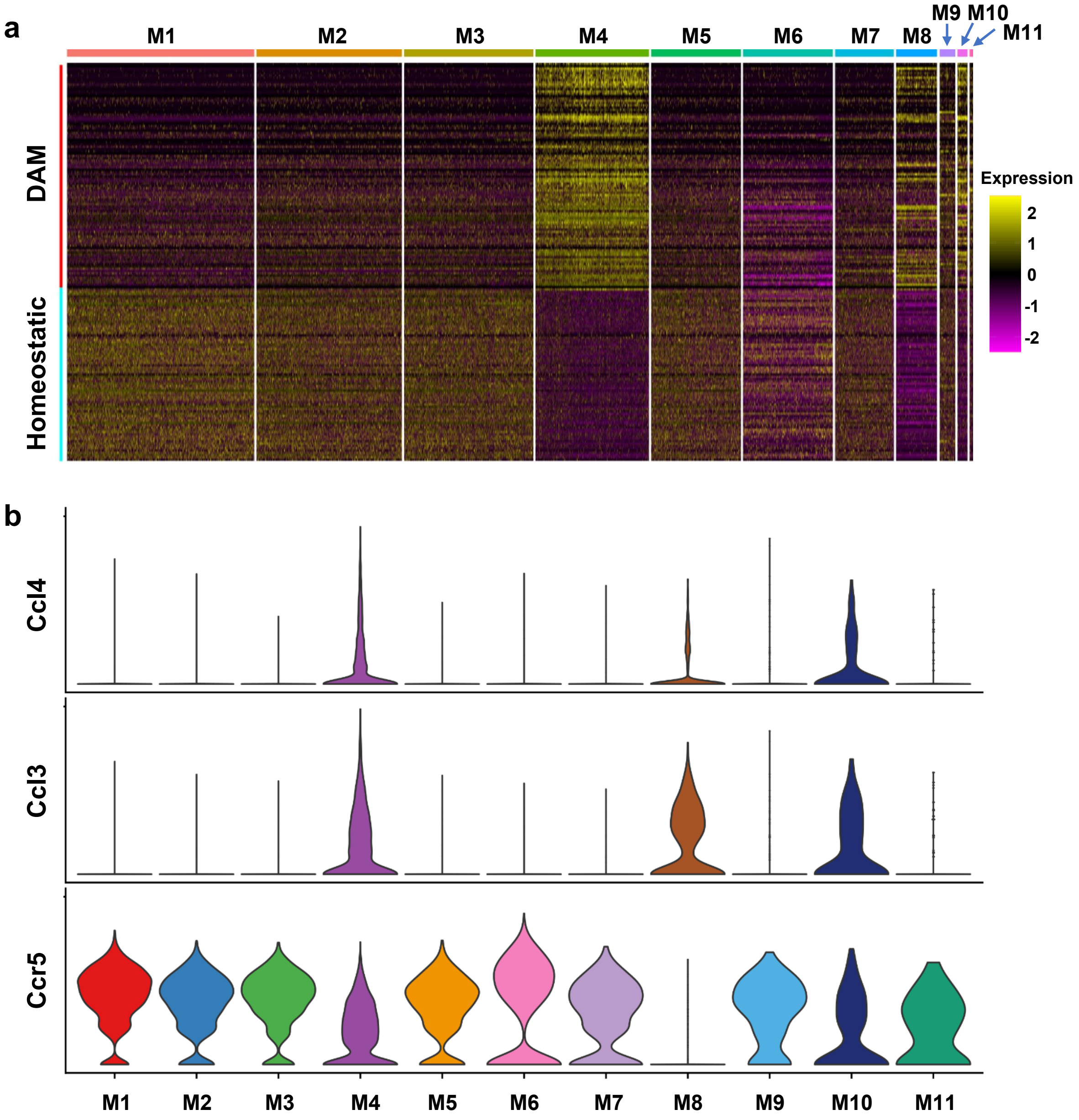


**Supplementary Fig. 10 | Expression of DAM and Homeostatic genes in microglia clusters and their communication. a,** Heatmaps showing expression of DAM and homeostatic genes identified by Keren-Shaul *et al*., 2017 across microglia clusters 1-11. Expression is shown as standard deviations above (yellow) or below (purple) the scaled mean of expression of all genes for each cell. **b,** Violin plots show the expression of CCL receptor (*Ccr5*) and ligands (*Ccl3* and *Ccl4*) across microglial clusters. Source data are provided as Supplementary information.
