# Supplementary material for: Effects of SPI1-mediated transcriptome remodeling on Alzheimer’s disease-related phenotypes in mouse models of Aβ amyloidosis
Source: Nat Commun. 2024 May 11;15:3996. doi: 10.1038/s41467-024-48484-x (PMC11088624; doi:10.1038/s41467-024-48484-x)
Supplement: Supplementary file 3 — Description of Additional Supplementary Files [file 41467_2024_48484_MOESM3_ESM.pdf]

File Name: Supplementary Data 1

Description: nCounter® Mouse AD Panel - Gene list

File Name: Supplementary Data 2

Description: *Spi1*$^{+/-}$;APPPS1 vs APPPS1 - NanoString list

File Name: Supplementary Data 3

Description: *Spi1*$^{+/-}$;APPPS1 vs APPPS1 - Pathway enrichment analysis using the Enrichr software

File Name: Supplementary Data 4

Description: *Spi1*$^{+/-}$;APPPS1 vs APPPS1 - Pathway enrichment analysis using the MetaCore software

File Name: Supplementary Data 5

Description: *Spi1*$^{TG/0}$;5xFAD vs 5xFAD - NanoString list

File Name: Supplementary Data 6

Description: *Spi1*$^{TG/0}$;5xFAD vs 5xFAD - Pathway enrichment analysis using the Enrichr software

File Name: Supplementary Data 7

Description: *Spi1*$^{TG/0}$;5xFAD vs 5xFAD - Pathway enrichment analysis using the MetaCore software

File Name: Supplementary Data 8

Description: LIMMA-Transcription Factors enrichment analysis

File Name: Supplementary Data 9

Description: LIMMA-Pathway enrichment analysis

File Name: Supplementary Data 10

Description: LIMMA-GO processes enrichment analysis

File Name: Supplementary Data 11

Description: LIMMA-Process Network enrichment analysis

File Name: Supplementary Data 12

Description: Cell-type clusters DEGs between *Spi1*$^{Tg/0}$;5XFAD and 5XFAD mice

File Name: Supplementary Data 13

Description: Ontology of Microglial clusters DEGs (Biological Process, BP; Molecular Function, MF; Cellular Component, CC; KEGG)

File Name: Supplementary Data 14

Description: Modules of scWGCNA (network edge and weight)

File Name: Supplementary Data 15

Description: CellChat Comparion between *Spi1*$^{Tg/0}$;5XFAD and 5XFAD mice

File Name: Supplementary Data 16

Description: Sequence of the primers used in the study
