# Supplementary material for: Effects of SPI1-mediated transcriptome remodeling on Alzheimer’s disease-related phenotypes in mouse models of Aβ amyloidosis
Source: Nat Commun. 2024 May 11;15:3996. doi: 10.1038/s41467-024-48484-x (PMC11088624; doi:10.1038/s41467-024-48484-x)
Supplement: Supplementary file 20 — Reporting Summary [file 41467_2024_48484_MOESM20_ESM.pdf]

nature portfolio

Corresponding author(s): Jungsu Kim

Last updated by author(s): Mar 20, 2024

Reporting Summary

Nature Portfolio wishes to improve the reproducibility of the work that we publish. This form provides structure for consistency and transparency in reporting. For further information on Nature Portfolio policies, see our Editorial Policies and the Editorial Policy Checklist.

Statistics

For all statistical analyses, confirm that the following items are present in the figure legend, table legend, main text, or Methods section.

n/a	Confirmed	
☐	☒	The exact sample size (*n*) for each experimental group/condition, given as a discrete number and unit of measurement
☐	☒	A statement on whether measurements were taken from distinct samples or whether the same sample was measured repeatedly
☐	☒	The statistical test(s) used AND whether they are one- or two-sided<br>*Only common tests should be described solely by name; describe more complex techniques in the Methods section.*
☒	☐	A description of all covariates tested
☐	☒	A description of any assumptions or corrections, such as tests of normality and adjustment for multiple comparisons
☐	☒	A full description of the statistical parameters including central tendency (e.g. means) or other basic estimates (e.g. regression coefficient) AND variation (e.g. standard deviation) or associated estimates of uncertainty (e.g. confidence intervals)
☐	☒	For null hypothesis testing, the test statistic (e.g. *F*, *t*, *r*) with confidence intervals, effect sizes, degrees of freedom and *P* value noted<br>*Give P values as exact values whenever suitable.*
☒	☐	For Bayesian analysis, information on the choice of priors and Markov chain Monte Carlo settings
☒	☐	For hierarchical and complex designs, identification of the appropriate level for tests and full reporting of outcomes
☐	☒	Estimates of effect sizes (e.g. Cohen's *d*, Pearson's *r*), indicating how they were calculated

Our web collection on statistics for biologists contains articles on many of the points above.

Software and code

Policy information about availability of computer code

Data collection	All images were obtained with a digital pathology slide scanner (Aperio VERSA; LEICA Biosystems) or inverted fluorescence microscope (DMi8; Leica Biosystems), For MSD Aβ assay data was acquired using MESO QuickPlex SQ 120 (multiplexing imager, MSD). For NanoString data was acquired using nCounter System (NanoString). WB images were obtained with Amersham Imager 680 (GE healthcare), Quantitative real-time PCR data was acquired using QuantStudio 3 (Thermo Fisher).
Data analysis	N/A

For manuscripts utilizing custom algorithms or software that are central to the research but not yet described in published literature, software must be made available to editors and reviewers. We strongly encourage code deposition in a community repository (e.g. GitHub). See the Nature Portfolio guidelines for submitting code & software for further information.

Data

Policy information about availability of data

All manuscripts must include a data availability statement. This statement should provide the following information, where applicable:

- Accession codes, unique identifiers, or web links for publicly available datasets
- A description of any restrictions on data availability
- For clinical datasets or third party data, please ensure that the statement adheres to our policy

Imaging data were processed and analyzed using ImageJ (1.53f). Data were analyzed using MSD Discovery Workbench (for MSD Aβ assay), QuantStudio 3 (for qPCR),

ImageQuant™ (for WB). For NanoString data were analyzed nSolver Advanced analysis, RStudio (v1.2.5033), LIMMA (v3.40.6), EnrichR, Metacore, and STRING database (v11.0). For Single-Cell RNA sequencing data were analyzed using Rstudio (v1.4.1717), Seurat (v4.0.1), Cytoscape (v3.8.0), and CellChat (v1.1.2).

Data availability
The scRNA-seq data generated in this study have been deposited in the GEO database (GSE222624). The NanoString data (GSE225669) (Tables S2 and S5) was included in the Supplementary Information of this paper. The enrichments data generated in this study are provided in the Supplementary Information/Source Data file. Source data are provided with this paper.

Research involving human participants, their data, or biological material

Policy information about studies with human participants or human data. See also policy information about sex, gender (identity/presentation), and sexual orientation and race, ethnicity and racism.

Reporting on sex and gender	N/A
Reporting on race, ethnicity, or other socially relevant groupings	N/A
Population characteristics	N/A
Recruitment	N/A
Ethics oversight	N/A

Note that full information on the approval of the study protocol must also be provided in the manuscript.

Field-specific reporting

Please select the one below that is the best fit for your research. If you are not sure, read the appropriate sections before making your selection.

☒ Life sciences ☐ Behavioural & social sciences ☐ Ecological, evolutionary & environmental sciences

For a reference copy of the document with all sections, see nature.com/documents/nr-reporting-summary-flat.pdf

Life sciences study design

All studies must disclose on these points even when the disclosure is negative.

Sample size	Sample sizes were determined based on our previous studies and similar studies in the literature (PMID: 26582899, PMID: 20005821, 24893973)
Data exclusions	No data were excluded from any analysis performed. Or No data was excluded from the analysis.
Replication	Immunostaining was performed on three sections from each mouse. The average of the results from three sections was used to represent a data point for each mouse. MSD Aβ assay included N=2 technical replicates and we presented the average value from two replicates. The results are consistent and robust.<br>The Aβ uptake assay comprised N=5 per group and was independently repeated twice. The repeated outcomes consistently demonstrate robustness.
Randomization	The order of animals in the staining experiments was randomized for each staining to minimize potential effects of systemic staining issues.<br>The qPCR analysis in Figures 5d and 5h was performed randomly with eight mice per group.<br>The WB analysis in Supplementary Fig 3 was performed randomly with six mice per group.<br>Throughout the other experiment, we prioritized using biological samples from all animals for analysis. However, for NanoString analysis, we strategically selected three mice per group closest to the mean Aβ42 accumulation levels to ensure representative data.
Blinding	All the experiment and analysis were performed blindly.

Reporting for specific materials, systems and methods

We require information from authors about some types of materials, experimental systems and methods used in many studies. Here, indicate whether each material, system or method listed is relevant to your study. If you are not sure if a list item applies to your research, read the appropriate section before selecting a response.

Materials & experimental systems

n/a	Involved in the study
☐	☒ Antibodies
☐	☒ Eukaryotic cell lines
☒	☐ Palaeontology and archaeology
☐	☒ Animals and other organisms
☒	☐ Clinical data
☒	☐ Dual use research of concern
☒	☐ Plants

Methods

n/a	Involved in the study
☒	☐ ChIP-seq
☒	☐ Flow cytometry
☒	☐ MRI-based neuroimaging

Antibodies

Antibodies used

Primary antibodies: For histology analysis, we used mouse Anti-Amyloid β (82E1) (1:500, IBL10323; IBL-AMERICA), rabbit Anti-IBA1 (1:1000 for cryosections and 1:400 for paraffin sections, ab178846; abcam), rabbit Anti-GFAP (1:1000, Z0334; Agilent Dako), rabbit Anti-LAMP1 (1:200, ab24170; abcam). For Western blot assay, we used rabbit anti-β-Amyloid precursor protein (βAPP, 1:500, #51-2700; Invitrogen), rabbit anti-β-secretase 1 (BACE1, 1:1000, #5606; Cell Signaling Technology), mouse Anti-Amyloid β (82E1) (to detect βCTF, 1:500, IBL10323), and mouse anti-β-actin (1:50,000, A1978; Sigma-Aldrich)
Secondary antibodies: Biotinylated goat anti-Mouse IgG (H+L) (1:400, BA-9200; Vector Laboratories), Alexa Fluor® 488 donkey Anti-Rabbit (1:500, 712-545-152; Jackson Laboratory), Alexa Fluor® 568 goat anti-Rabbit (1:500, A-11011; Invitrogen), goat anti-Rabbit IgG, HRP-conjugated (1:5,000, #7074, Cell Signaling Tech), donkey anti-Mouse IgG, HRP-conjugated (1:10,000, sc-2096; Santa Cruz)

Validation

1. Validation for immunohistochemistry by the company (companies' websites)
mouse Anti-Amyloid β (82E1) (IBL-AMERICA, https://www.ibl-america.com/amyloid-beta-n-82e1-a-anti-human-mouse-igg-moab-1/)
rabbit Anti-IBA1 (abcam, https://www.abcam.com/iba1-antibody-epr16588-ab178846.html),
rabbit Anti-GFAP (Agilent Dako, https://www.agilent.com/en/product/immunohistochemistry/antibodies-controls/primary-antibodies/glial-fibrillary-acidic-protein-%28concentrate%29-76683), rabbit Anti-LAMP1 (abcam, https://www.abcam.com/lamp1-antibody-lysosome-marker-ab24170.html).
2. Validation for Western blots by the company (companies' websites)
rabbit anti-β-Amyloid precursor protein (βAPP, Invitrogen, https://www.thermofisher.com/antibody/product/beta-Amyloid-Antibody-clone-CT695-Polyclonal/51-2700), rabbit anti-β-secretase 1 (BACE1, Cell Signaling Technology, https://www.cellsignal.com/products/primary-antibodies/bace-d10e5-rabbit-mab/5606), mouse Anti-Amyloid β (82E1) (to detect βCTF, https://www.ibl-america.com/amyloid-beta-n-82e1-a-anti-human-mouse-igg-moab-1/), and mouse anti-β-actin (Sigma-Aldrich, https://www.sigmaaldrich.com/US/en/product/sigma/a1978)
3. Validation for Secondary antibodies by the company (companies' websites)
Biotinylated goat Anti-Mouse IgG (H+L) (Vector Laboratories, https://vectorlabs.com/products/antibodies/biotinylated-goat-anti-mouse-igg), Alexa Fluor® 488 donkey Anti-Rabbit (Jackson Laboratory, https://www.jacksonimmuno.com/catalog/products/711-545-152), Alexa Fluor® 568 goat anti-Rabbit (Invitrogen, https://www.thermofisher.com/antibody/product/Goat-anti-Rabbit-IgG-H-L-Cross-Adsorbed-Secondary-Antibody-Polyclonal/A-11011), goat anti-Rabbit IgG, HRP-conjugated (Cell Signaling Tech, https://www.cellsignal.com/products/secondary-antibodies/anti-rabbit-igg-hrp-linked-antibody/7074), donkey anti-Mouse IgG, HRP-conjugated (Santa Cruz, https://datasheets.scbt.com/sc-2096.pdf)

Eukaryotic cell lines

Policy information about cell lines and Sex and Gender in Research

Cell line source(s)

BV-2 microglia cell line (RRID:CVCL_0182)

Authentication

Declare that the used cell line was not authenticated.

Mycoplasma contamination

No mycoplasma detected in tested cell line.

Commonly misidentified lines (See ICLAC register)

No commonly misidentified cell lines were used in the study.

Animals and other research organisms

Policy information about studies involving animals; ARRIVE guidelines recommended for reporting animal research, and Sex and Gender in Research

Laboratory animals

All mice were maintained under a 12-hour light/dark cycle in a temperature-controlled room with free access to food and water. All animal studies were approved and performed in compliance with the guidelines of the Institutional Animal Care and Use Committee of the Indiana University School of Medicine (Protocol ID: 21149).
PU.1-knockdown (KD) mice (Spi1+/−, C57BL6 background, Strain #:006083) and littermate control PU.1-wildtype (WT) mice were sacrificed at 2 months of age.
PU.1-transgenic (TG) (Spi1Tg/0, B6;FVB background, Strain #:006147) mice and littermate control PU.1-wildtype (WT) mice were sacrificed at 2 months of age.
APP/PS1;PU.1 KD mice and littermate control APP/PS1;PU.1 WT mice were sacrificed at 4 months of age.

	5XFAD (#034840-JAX);PU.1 TG mice and littermate control 5XFAD;PU.1 NTG mice were sacrificed at 4 months of age.
Wild animals	This study did not involve the use of any wild animals.
Reporting on sex	The APP/PS1 mouse model is known to have no sex effect on Aβ levels and amyloid deposition, and our data also demonstrated no differential sex effects on Aβ accumulation (Extended Data Figure 2a-d). Total 16 males and 12 females mice were used.<br>5XFAD mouse model has a significant sex difference in Aβ levels and amyloid deposition, we performed all statistical analyses for male and female separately. Total 21 males and 19 females mice were used.
Field-collected samples	The study did not involve the samples collected from the field.
Ethics oversight	The study received ethical approval from IUSM Institutional Animal Care and Use Committee. All mice procedures were performed in accordance with the Guide for the Care and Use of Laboratory Animals (National Institutes of Health, Bethesda, MD). Mice were euthanized according to IUSM Institutional Animal Care and Use Committee-approved procedures.

Note that full information on the approval of the study protocol must also be provided in the manuscript.

Plants

Seed stocks	N/A
Novel plant genotypes	N/A
Authentication	N/A
